# Tumor deposits on MRI in rectal cancer—detection and differentiation from lymph nodes with histopathological correlation

**DOI:** 10.1007/s00330-026-12467-x

**Published:** 2026-03-18

**Authors:** Simon Lundström, Staffan Täckström, Peder Wiklund, Maja Haskå, Erik Agger, Lennart Nedar, Pamela Buchwald

**Affiliations:** 1 Department of Surgery, Skåne University Hospital, Malmö, Sweden; 2 Department of Clinical Sciences, Lund University, Lund, Sweden; 3 Department of Radiology, Central Hospital Västerås, Västerås, Sweden; 4 Department of Surgical Sciences, Uppsala University, Uppsala, Sweden; 5 Department of Radiology, Region Halland, Halmstad, Sweden; 6 Department of Pathology, Skåne University Hospital, Malmö, Sweden; 7 Department of Nuclear Medicine and Radiation Physics, Karolinska University Hospital, Stockholm, Sweden; 8 Department of Molecular Medicine and Surgery, Karolinska Institutet, Stockholm, Sweden

**Keywords:** Rectal neoplasms, Magnetic resonance imaging, Extranodal extension, Lymph nodes, Diagnostic accuracy

## Abstract

**Objective:**

Tumor deposits (TDs) are a negative prognostic factor in colorectal cancer, independent of lymph node metastasis (LNM). This study aimed to investigate the ability of pre-operative MRI to identify TDs and differentiate them from LNM in rectal cancer.

**Materials and methods:**

Patients with histopathological N1-N2 rectal cancer with and without TDs who underwent curative-intent primary surgery between 2013 and 2023 were retrospectively included. Three radiologists independently reviewed pre-operative MR images for the presence of TDs and LNM. Histopathology served as the reference standard from which interobserver agreement by Cohen’s kappa (κ) was calculated. Unblinded node-to-node comparison of MRI and histopathological images was used to identify sources of discrepancies between the modalities. Post hoc analysis stratified on nodal size (> or ≤ 5 mm) was performed.

**Results:**

Eighty-eight patients (50% with TDs on histopathology, 74% male, median age 70 [20–89] years) were included, of whom 27 were excluded due to poor image quality. Agreement between MRI and histopathology (p), both for TD-presence (κ = −0.04) and LNM-presence (κ = 0.16), was low. In the node-by-node comparison, 43 out of 50 pTDs (86%) measured ≤ 5 mm, and 131 of 167 pLNM (78%) measured ≤ 5 mm. In post hoc analysis of smaller (≤ 5 mm) nodules, no pTDs and 3% of pLNM were diagnosed using MRI.

**Conclusion:**

MRI demonstrated limited ability in detecting and differentiating TDs and LNM in pre-operative low-risk patients. Diagnostic performance was particularly poor for nodules ≤ 5 mm, which constituted 174 out of 217 (80%) pathological nodular structures.

**Key Points:**

***Questions*** *Tumor deposits and lymph node metastasis are important factors for staging rectal cancer, but the ability of pre-operative MRI to detect and differentiate them remains unclear*.

***Findings*** *In low-risk rectal cancer patients treated with primary surgery, tumor deposits and lymph node metastasis are often small (≤ 5 mm) and not detected by MRI*.

***Clinical relevance*** *MRI has a limited ability to identify and differentiate small (≤ 5 mm) tumor deposits and lymph node metastasis in rectal cancer, which must be considered during pre-operative staging and neoadjuvant treatment planning*.

**Graphical Abstract:**

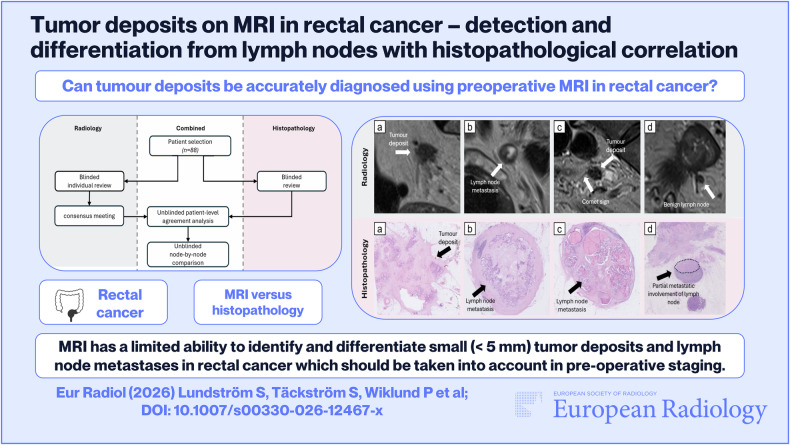

## Background

Tumor deposits (TDs) are a known negative prognostic factor for overall survival and distant metastasis in both colon and rectal cancer, present in 20% of cases [1–4]. Magnetic resonance imaging (MRI) is used for pre-operative staging of rectal cancer; however, TDs are mainly diagnosed postoperatively through histopathological examination of the resected specimen (pTD), alongside assessment of lymph node metastasis (pLNM) [5].

TDs are defined histopathologically as discontinuous nodules of cancer cells without any vascular, neural or lymphatic tissue [5]. On MRI, both TDs and LNM are seen as nodal structures. The radiological classification of LNM follows the ESGAR expert consensus guidelines, where the combination of size and morphology is used [6]. TDs lack a clear definition but are usually nodular, separate from the primary tumor, vessel-associated (comet sign), and morphologically distinct from LNM [7].

Since the prognostic importance of TDs has been increasingly recognized, pretreatment detection and quantification of the number of TDs using imaging has gained increasing interest [1]. Lord et al demonstrated that TDs can be identified preoperatively using MRI (mrTD) with a high interobserver agreement (κ > 0.77) [8]. In the same study, they suggested that mrLNM, in contrast to TNM, does not constitute an independent risk factor when distinguished from mrTDs, which may be associated with the hypothesized, more aggressive, vascular metastatic pathway of TDs [8, 9].

Earlier diagnosis and the ability to distinguish pTDs from pLNM preoperatively could enhance risk stratification and optimize selection for targeted neoadjuvant treatment, potentially improving long-term oncologic outcomes for colorectal cancer patients [10]. In retrospective material, TDs seem to have a lesser response to current neoadjuvant therapy compared to LNM, although the causality remains to be proven [11]. However, no studies have validated mrTD compared to pTD in patients who have not received neoadjuvant treatment, thereby enabling a direct comparison between MRI and histopathology.

This study aimed to quantify the diagnostic accuracy of MRI in identifying TDs and differentiating them from LNM in patients with primary surgery for rectal cancer, using node-by-node histopathological correlation.

## Patients and methods

### Study population

**Fig. 1:**
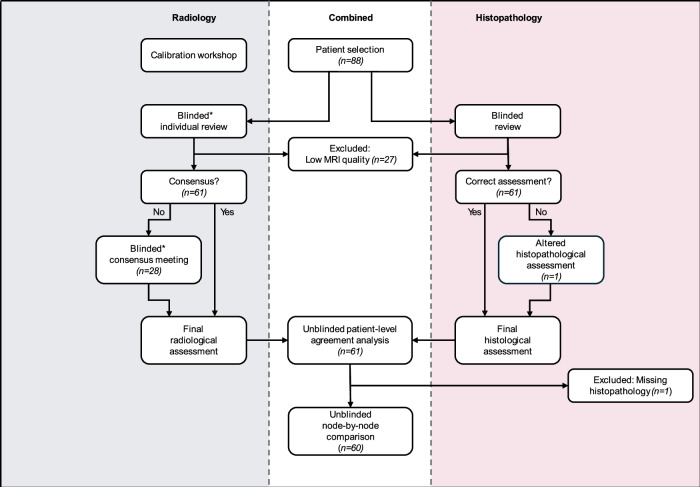
Overview of the study workflow. Patient selections were performed using the Swedish Colorectal Cancer Registry. MR images were independently reviewed by three radiologists in blinded sessions, followed by a blinded* consensus meeting when required. Histopathological re-review was conducted independently according to TNM8. Final agreement was established through unblinded patient-level agreement analysis. Diagnostic discrepancies were investigated through node-by-node comparison between MRI and histopathology. Numbers (*n = xx*) indicate the number of patients included at each stage of the review process. Background colors symbolize imaging modalities: gray = magnetic resonance imaging workflow; pink = histopathological workflow. * Radiologists blinded to the histopathological results

All patients with rectal cancer who had undergone curative-intent primary (i.e., without neoadjuvant treatment) surgical resection of the rectum at Skåne University Hospital, Scania, Sweden, between 2013 and 2023 were retrospectively localized using regional data from the Swedish Colorectal Cancer Registry (SCRCR). Rectal cancer was defined as adenocarcinoma with the lower border located within 15 cm from the anal verge measured with rigid sigmoidoscopy. Patients who had missing registered pTD status or had received neoadjuvant treatment were excluded. All pTD-positive patients were identified and matched with pTD-negative patients by sex, pT-stage, pN-stage, and age on a 1:1 ratio to serve as controls. Data on patient characteristics and histopathology were extracted. The study workflow is summarized in Fig. 1.

### Magnetic resonance imaging

All MR examinations were prospectively carried out as part of routine clinical workup. All examinations were performed in the Scania region, using a broad range of MRI systems from three different vendors operating at either 1.5 T or 3 T. Detailed information about scanners and imaging parameters is presented in Supplement 1. Interpretation for the study was primarily based on T2-weighted images using all three planes with an in-plane resolution ranging from 0.5 × 0.6 to 1.0 × 1.0 mm (maximum 0.98 × 0.98 mm). Patients were routinely prescribed a micro-enema to take 2–4 h prior to the examination. Intravenous or rectal contrast agents were not part of the clinical routine and were therefore not included in the analysis.

### Radiological assessment

MR images were retrospectively retrieved, pseudonymized and uploaded to a web-based zero footprint DICOM viewer (www.cmrad.com, Collective Minds AB). Imaging analysis was performed by three independent radiologists: L.N. (30 years of experience), P.W. (12 years of experience) and S.T. (13 years of experience). Prior to the review, L.N. conducted a calibration workshop with all participating radiologists to ensure MR-imaging criteria consistency. Radiology review was blinded to histopathology and reported according to a predetermined form where each nodal structure was assessed and marked on a 3D-matrix (Supplement 2). Lymph node assessment was performed according to Swedish guidelines based on the 2016 ESGAR expert consensus guidelines, where nodes with a short-axis diameter of < 5 mm, 5–9 mm, and ≥ 9 mm require 3, 2, and 1 malignant features, respectively (irregular border, internal heterogeneity, and round shape) [6]. Mucinous nodes were considered malignant [6, 12]. In this study, free nodular structures in the mesorectum were assessed as TDs if they lacked clear lymph node morphology and showed distinctly irregular spiculated borders, regardless of size or presence of comet sign.

After the review, cases with low MR-image quality due to insufficient spatial resolution or artefacts obviating assessment were excluded. For the remaining patients, a second joint consensus meeting determined the presence of mrTD and mrLNM and established agreement in each case.

### Histopathology

The existing histopathological assessments of the surgical specimen served as reference standard. During the early part of the study period, assessments were made according to TNM7, and from 2017 onward, TNM8. The criteria for TDs changed between these versions. In both versions, pTDs were defined as foci of tumor cells discontinuous from the primary tumor, located within the lymphatic drainage of the perirectal fat tissue without evidence of lymphatic tissue [13]. With the introduction of TNM8, the criteria for TDs required that no remaining vascular or neural tissue would be present around the tumor; otherwise, these foci would be classified as EMVI [5, 13]. A single pathologist (M.H., 12 years of experience) re-reviewed the digital histopathological images for all cases according to TNM8, blinded for the radiological consensus.

### Radiological–histopathological unblinded correlation

Following consensus review, a meeting was arranged where the three participating radiologists and the pathologist had MR images and digital histopathological slides available. A node-by-node comparison between MRI and histopathology was performed to explore sources of discrepancies between MRI and histopathology, potentially being (1) size of histopathological finding being too small to identify on MRI (2) identification of the structure on MRI but misclassification for other reason than size compared to histopathology (3) other source of lack of identification/misclassification on MRI such as area not examined with MRI or histopathology.

**Fig. 2:**
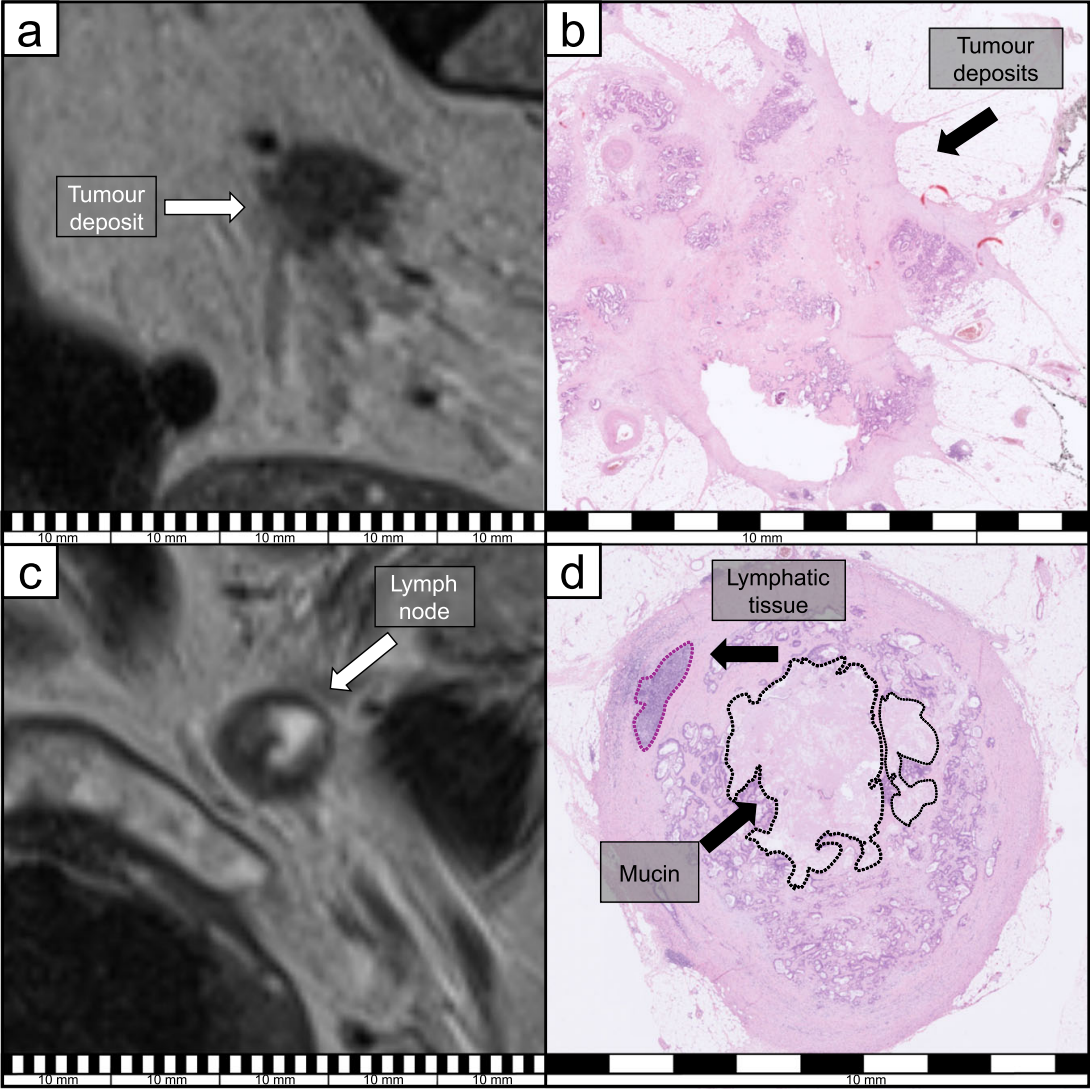
Example of matched lesions during the radiological–histopathological correlation process. **a** Sagittal T2-weighted image with a 10 mm tumor deposit with irregular spiculated borders, with (**b**) corresponding magnified histopathological image of a tumor deposit. **c** Sagittal T2-weighted MR image showing a 10 mm lymph node metastasis (white arrow) with central high signal, with (**d**) corresponding histopathological image of a nodal structure with central mucin (dotted black line) and a small area of lymphatic tissue (dotted purple line), making it a lymph node metastasis instead of a tumor deposit. Note the varying scale between the images

Nodal MRI findings were cross-referenced with histopathological slides. To ensure that the same structure was evaluated on both microscopy and MRI, the size, shape (assessed by both radiologists and the pathologist), proximity to the primary tumor (when present on the same microscopic section), and proximity to the resection margin (when color-marked on the pathology image) were used as indicators to achieve the most accurate possible node-by-node comparison (Fig. 2). Discrepancies were noted and classified per size (> 5 mm or ≤ 5 mm) and pathological nodal diagnosis (TD, LNM or EMVI) or, in cases where no corresponding nodal structures could be identified on the histopathological slides, as missing.

### Statistical analysis

Numerical data were reported as median with interquartile range. Categorical variables were presented as numbers and proportions in percentages. Sensitivity and specificity for prevalence on a patient level (yes/no) were calculated with Clopper–Pearson confidence intervals. Cohen’s kappa analysis was performed to assess agreement between MRI-variables (mrTD and mrLNM) and their histopathological equivalent (pTD and pLNM).

Following unblinded radiological–histopathological correlation, a post hoc analysis was performed where nodal structures were stratified by size (> 5 mm or ≤ 5 mm on short-axis MRI) and cross-tabulated against the histopathology assessments. Because information on true-negative nodal structures was unavailable, only sensitivity and positive predictive value were calculated for mrTD and mrLNM in nodal structures > 5 mm. For sensitivity analyses, a misclassification of a pTD as mrLNM (or vice versa) was considered a false negative.

All statistical analyses were performed using R version 4.3.1 (R Core Team, 2023) with RStudio 2024.12.1 (RStudio Team, 2020). A Cohen’s Kappa value of less than 0.20 was interpreted as non-existent agreement, and a value of over 0.9 as complete agreement [14].

### Ethical considerations

The study was approved by the Swedish Ethical Review Authority (DNR: 2023-06664-02). Patients were identified using existing regional data from SCRCR.

## Results

### Patient cohort and baseline characteristics

Between 2013 and 2022, a total of 44 TD-positive rectal cancer patients who had undergone primary surgery were identified within the registry, for which 44 matching TD-negative patients were identified. After image retrieval, 27 patients (15 pTD-positive and 12 pTD-negative) were excluded due to poor image quality. In addition to limited in-plane spatial resolution (> 1 × 1 mm), exclusions were mainly due to rectosigmoid tumors, either affected by motion artifacts or insufficient coverage of the mesorectum near the cranial tumor margin or adjacent nodular structures.

Baseline clinical characteristics of the remaining 61 patients are presented in Table 1. The majority of patients were male (74%) with a median age of 70 [20–89] years. Patient characteristics and histopathological findings, including pLNM count, were comparable between the groups, except for EMVI, which was more frequent in pTD-positive patients (44%) compared to pTD-negative patients (34%). Because we only included patients who had not receive neoadjuvant therapy, most tumors were staged mrT3b or lower. However, some mrT3c/d and limited mrT4 tumors were included, as high rectal cancers may undergo upfront surgery according to national guidelines [12]. The median time from MRI to surgery was 34 [27–43] days for pTD-positive patients and 31 [26–45] days for pTD-negative patients.

**Table 1:** Baseline characteristics for pTD-positive and pTD-negative rectal cancer patients

	pTD-negative (*N* = 32)	pTD-positive (*N* = 29)	Total (*N* = 61)
Sex (male)	24 (75.0)	21 (72.4)	45 (73.8)
Age (years)	71 [44–89]	69 [20–83]	70 [20–89]
BMI (kg/m^2^)	26 [24–27]	26 [23–28]	26 [23–28]
mrT-stage			
T1–T2	9 (28.1)	10 (34.5)	19 (31.1)
T3	21 (65.6)	18 (62.1)	39 (63.9)
T4	1 (3.1)	0 (0)	1 (1.6)
TX	1 (3.1)	1 (3.4)	2 (3.3)
mrN-stage			
N0	13 (40.6)	16 (55.2)	29 (47.5)
N1–2	19 (59.4)	13 (44.8)	32 (52.5)
pT-stage			
T1	0 (0)	1 (3.4)	1 (1.6)
T2	1 (3.1)	3 (10.3)	4 (6.6)
T3	26 (81.3)	23 (79.3)	49 (80.3)
T4	5 (15.6)	2 (6.9)	7 (11.5)
pN-stage			
N1	24 (75.0)	22 (75.9)	46 (75.4)
N2	8 (25.0)	7 (24.1)	15 (24.6)
pLymph node metastases	2 [1–4]	2 [1–3]	2 [1–3]
pTumor deposit count	0 [0–0]	1 [1–2]	0 [0–1]
pEMVI (yes)	11 (34.4)	13 (44.8)	24 (39.3)
Mucinous tumor (yes)	3 (9.4)	3 (10.3)	6 (9.8)

Continuous values are presented as median [interquartile range]. Categorical values are presented as frequency (%)

*c* clinical, *p* histopathological, *EMVI* extramural vascular invasion

### Radiological review and histopathological reclassification

Of the remaining 61 patients, 28 (46%), of whom 50% were pTD-positive, did not reach radiological consensus and required discussion at a consensus meeting. Discrepancies were related to the assessment of mrTD count in 11% of cases, mrLNM count in 39%, and both parameters in 50%.

Histological examination prior to the radiological–histopathological agreement analysis was performed to ensure consistency with, and adherence to, TNM8. One pTD-negative/pLNM-positive patient was changed to pTD-negative/pLNM-negative as no pLNM were identified. Additionally, a patient with pT3N2bM0 tumor was excluded as it lacked complete histological slides.

### Patient-level agreement between radiology and histopathology

Interpatient comparison between the consensus MRI review and the reported histopathology on a patient level (i.e., mrTD yes/no) revealed very low agreement between mrTDs and pTDs (κ = −0.04, Table 2), with MRI showing low diagnostic sensitivity (21%, 95% CI: 8–40%) and average specificity (75%, 95% CI: 57–89%). Similarly, the agreement between mrLNM and pLNM was very low (κ = 0.14, Table 3), with low sensitivity (41%, 95% CI: 28–55%) but high specificity (100%, 95% CI: 59–100%).

**Table 2:** Agreement between MRI and histopathology for the detection of TDs on a patient level

Tumor deposits	mrTD+	mrTD−
pTD+	6	23
pTD−	8	24

Rows represent the number of patients with positive or negative MR findings, whereas columns represent histopathological findings

Cohen’s Kappa agreement = −0.0442 (Very low)

*TD* tumor deposits, *p* histopathological, *mr* magnetic resonance

**Table 3:** Agreement between MRI and histopathology for the detection of LNM on a patient level

Lymph node metastasis	mrLNM+	mrLNM−
pLNM+	22	32
pLNM−	0	7

Rows represent the number of patients with positive or negative MR findings, whereas columns represent histopathological findings

Cohen’s Kappa agreement = 0.157 (Very low)

*LNM* lymph node metastasis, *p* histopathological, *mr* magnetic resonance

### Node-by-node correlation between radiology and histopathology

Node-by-node matching between MRI and histopathology was feasible primarily for nodal structures > 5 mm, as smaller pathological foci could not be reliably identified or confirmed on MRI and were therefore often assessed as mrTD− or mrLNM-negative. To account for this limitation, a post hoc analysis was performed, stratifying nodular structures by short-axis length (> 5 mm or ≤ 5 mm) on MRI (Table 4).

**Table 4:** Nodal agreement between MRI and histopathology diagnostics based on size

Pathological nodal structures	Total	mrLNM+	mrTD+	mrNeg
pLNM+	**167**	**26**	**10**	**131**
* pLNM*+ *> 5*	*36/167 (22%)*	*22/26 (85%)*	*7/10 (70%)*	*7/131 (5%)*
* pLNM*+* ≤ 5*	*131/167 (78%)*	*4/26 (15%)*	*3/10 (30%)*	*124/131 (95%)*
pTD*+*	**50**	**0**	**3**	**47**
* pTD*+* > 5*	*7/50 (14%)*	*-*	*3/3 (100%)*	*(2* + 2*)/47 (9%)
* pTD*+* ≤ 5*	*43/50 (86%)*	*-*	*0/3 (0%)*	*43/47 (91%)*
pNeg	**-**	**4**	**2**	**-**

Rows represent the number of positive nodal MR findings, whereas columns represent the number of positive nodal histopathological findings. Bold rows are constituted of the primary comparison, whereas cursive rows are divided into size based on the post hoc analysis

*TD* tumor deposits, *LNM* lymph node metastasis, *p* histopathological, *mr* magnetic resonance

* In two patients, a nodal MR structure > 5 mm could be identified in the side-by-side comparison, whereas two had no identifiable MRI finding

In the node-by-node comparison, 43 out of 50 TDs (86%) measured ≤ 5 mm, and of 167 LNM, 131 (78%) measured ≤ 5 mm on histopathology. Among pTDs missed by MRI, 91% were ≤ 5 mm on histopathology, whereas the corresponding proportion for pLNM was 95%. No pTD ≤ 5 mm were accurately identified as a mrTD, whereas only 3% of pLNM ≤ 5 mm were identified as mrLNM. pLNM was assessed as mrTD in 6% of nodules, whereas no pTD was classified as mrLNM. As expected, the total number of nodal structures exceeded the number of patients with mrTD or mrLNM, as demonstrated in Tables 2 and 3.

For nodular structures > 5 mm, MRI had a sensitivity of 43% (95% CI: 0.10–0.82) for pTD and 61% (95% CI: 0.44–0.77) for pLNM. Among nodal structures > 5 mm, MRI erroneously classified two benign nodal structures as mrTD, yielding a nodal positive predictive value for mrTD of 60% (95% CI: 0.15–0.95), whereas four benign nodal structures were misclassified as mrLNM, yielding a nodal positive predictive value for mrLNM of 85% (95% CI: 0.65–0.96).

## Discussion

This study contributes to the limited evidence on the diagnostic accuracy of pre-operative assessment of TDs on MRI against the histopathological reference standard. To our knowledge, this is the first node-by-node validation in rectal cancer patients without neoadjuvant treatment aiming to preoperatively identify TDs. The cohort represents a clinically relevant group where the detection of TDs could influence management by prompting neoadjuvant therapy. The unblinded, side-by-side comparison of MRI and histopathology suggests that, although larger nodal structures (> 5 mm) may be identifiable on MRI, small (≤ 5 mm) TDs and LNMs are common (86% and 78%, respectively) and fall below the reliable detection and characterization threshold of MRI standards in our study. The difficulty in diagnosing and distinguishing small TDs from LNMs using MRI calls for awareness in the pre-operative setting. Three experienced radiologists from different Swedish centers independently reviewed all MRI cases after a calibration workshop to improve consistency. Despite this, nearly half of the cases required a consensus meeting to resolve discrepancies, highlighting challenges associated with the interpretation of MRI in rectal cancer. The initial radiological–histopathological review revealed low interobserver agreement and low agreement for both TDs and LNM, which is in contrast with the findings by Lord et al, who reported high interobserver agreement and high sensitivity for mrTD [8]. Besides different inclusion criteria for the study cohorts, several methodological differences may account for this.

First, Lord et al compared mrTD to the pathology reports without any histopathological re-review or node-by-node matching, including patients from as early as TNM5, where all nodal structures larger than 3 mm were reported as LNM, as the distinction with TDs was not yet defined. Second, our criteria for defining mrTDs differed from those used by Lord et al, who emphasized the comet tail sign, consisting of the inability to distinguish a TD from a vein in two projections [15]. This sign likely improves interobserver agreement but may reduce sensitivity, especially for small TDs with tiny veins that are difficult to visualize on MRI. Therefore, we did not exclusively apply this criterion as an invariable co-occurrence of comet signs for TDs is yet to be proven. Third, nearly all TD-positive patients in Lord et al’s cohort exhibited EMVI, which, according to Swedish national guidelines, typically leads to neoadjuvant treatment and thus exclusion from our study. Lastly, MRI data were obtained from multiple scanner vendors and acquisition protocols. While this heterogeneity reflects real-world clinical practice, it may have contributed to reduced diagnostic accuracy and differentiation performance.

The importance of identifying and differentiating TDs from LNM following surgery has been demonstrated by multiple studies, but effective treatment for TD-positive cancer patients is limited as accurate pre-operative diagnosis is lacking [1, 16, 17]. By investigating the diagnostic accuracy of MRI in identifying TDs and differentiating them from LNM prior to surgery, this study provides important methodological insights for future studies investigating the prognostic impact of neoadjuvant therapy on TD- and LNM-positive patients. However, the current inability to reliably differentiate TDs from LNMs on MRI limits the clinical utility of pre-operative nodal differentiation, particularly for small nodules ≤ 5 mm.

**Fig. 3:**
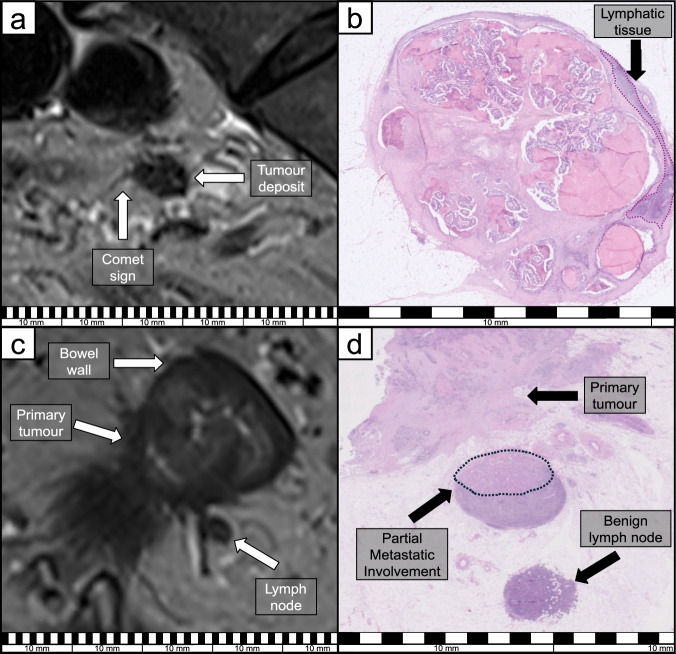
Example of diagnostic discrepancies identified during the radiological–histopathological correlation process. **a** T2-weighted MR image of an 11 mm irregular nodule, which was classified as a tumor deposit, note the presence of a vessel coursing into the lesion, judged as representing “comet sign.” In the corresponding histopathological image (**b**), lymphatic tissue is located at the nodal border (black arrow), making it a metastatic lymph node. **c** T2-weighted transversal MR image showing a well-circumscribed lymph node with a short-axis diameter of 4 mm that did not fulfill all necessary morphological criteria for metastasis and was therefore assessed as benign. On histopathology, a lymph node metastasis that matched in shape, size, and distance from the primary tumor node was identified, showing partial metastatic involvement (black dotted line and arrow) (**d**). Note the varying scale between the images

The challenge for MRI-based nodal staging in rectal cancer is well documented, with previous studies reporting low agreement with histopathology [7, 18–20]. The initial results in our study also showed poor agreement between TDs and LNMs. One reason may be that irregular borders were used as a malignant diagnostic criterion for LNM, in accordance with ESGAR, whereas TDs were assessed using similar, albeit stricter, criteria of “distinctly irregular spiculated borders.” Since this study was unable to reliably differentiate between mrTDs and mrLNM, the clinical utility of this distinction as a diagnostic criterion cannot be confirmed. Post hoc analysis also revealed difficulties in distinguishing TDs from LNM (Fig. 3a, b) and the high prevalence of small, metastatic areas within lymph nodes (Fig. 3c, d), likely contributing to this finding. These findings align with previous literature and further underscore the limitations of reliably identifying nodular metastases preoperatively using MRI [7]. In the current study, large nodal structures were more accurately identified, likely leading to higher sensitivity in more advanced high-risk tumors.

A major strength with the fact that patients had primary surgery is the node-to-node matching between diagnostic imaging and histopathological findings, mitigating any induced biologic confounding changes that may have occurred between MRI and pathology. This strength also introduced selection bias toward more early-stage tumors. However, for the pre-operative detection of TDs to be clinically meaningful, the potential to influence clinical management is a prerequisite. By excluding patients who had already received neoadjuvant treatment, the cohort was focused on those for whom TD identification could directly impact treatment decisions by flagging a high-risk feature but also a selection toward patients with potentially more limited and smaller TDs when present.

Our findings indicate that, in this clinically relevant patient group, the detection of TDs using standard MRI techniques is limited, likely due to the small size of the pTDs identified postoperatively. Major limitations were the retrospective design and the absence of up-to-date MR imaging, reflected in the high number of exclusions due to poor image quality. Finally, although the radiologists were blinded to histopathological results, the relatively high proportion of pTD-positive cases (50%) may have introduced bias toward overinterpreting nodal structures as TDs. Nevertheless, only 23% of patients were classified as TD-positive on MRI, underscoring the difficulty of identifying TDs in this population.

### Conclusion

In this preoperatively low-risk cohort of rectal cancer patients undergoing primary surgery, MRI demonstrated limited sensitivity and specificity in detecting and differentiating between TDs and LNM. Diagnostic performance was particularly poor for nodules ≤ 5 mm, which constituted 174 out of 217 (80%) of the pathological nodular structures. This study highlights the limitations of MRI for detecting and differentiating smaller TDs and LNM, which should be taken into account when interpreting and staging rectal cancer on MRI.

## Supplementary information

ELECTRONIC SUPPLEMENTARY MATERIAL

## Data Availability

The dataset analyzed during the current study is not publicly available due to sensitive information.

## Abbreviations

EMVI: Extramural vascular invasion
LNM: Lymph node metastasis
MRI: Magnetic resonance imaging
SCRCR: Swedish Colorectal Cancer Registry
TD: Tumor deposits
