# Supplementary material for: Tumor deposits on MRI in rectal cancer—detection and differentiation from lymph nodes with histopathological correlation
Source: Eur Radiol. 2026 Mar 18;36(8):6428–37. doi: 10.1007/s00330-026-12467-x (PMC13342202; doi:10.1007/s00330-026-12467-x)

Tumor deposits on MRI in rectal cancer – detection and differentiation from lymph nodes with histopathological correlation

ELECTRONIC SUPPLEMENTARY MATERIAL

Supplement 1

GE Medical Systems (Optima MR450w 1.5T, Discovery MR750w 3T, Signa Architect 3T), Philips Medical Systems (Intera 1.5T, Achieva dStream 1.5T, Intera 3T), and Siemens Healthineers (TrioTim 3T, Magnetom Vida 3T, Prisma 3T, Avanto Fit 1.5T, Symphony Tim 1.5T, Aera 1.5T, Avanto 1.5T) scanners were used. All examinations included T2-weighted images acquired in multiple planes without fat suppression. Imaging parameters varied across scanners. For the T2HR axial oblique series with slice thickness was 3-4 mm (two examinations were 4 mm; all others 3 mm), repetition time (TR) 2833–9280 ms, echo time (TE) 72–122 ms and in-plane resolution ranged from 0.5 x 0.6 to 1.0 x 1.0 mm (maximum 0.98 x 0.98 mm).

Analysis protocol for mrTD

Patient number:			Age:			
Date for MR			Sex:	**Male** ☐	**Female** ☐	
**mrTD**						
MR-quality	**Low** ☐	**High** ☐				
T-stage	**Tis** ☐	**T1** ☐	**T2** ☐	**T3** ☐	**T4a** ☐	**T4b** ☐
Tumor length (mm)						
Shortest tumor distance to MRF (mm)						
EMVI	**Yes** ☐	**No** ☐	**Number:**			
Mesorectal lymph nodes	**Total:**		**Positive:**			
Positive lymph node distance from lower tumor margin						
Shortest positive lymph node distance to MRF (mm)						
Positive mesorectal lymph node diameter (mm)	**Largest:**		**Smallest:**			
Positive mesorectal lymph nodes above the peritoneal reflection	**Yes** ☐	**No** ☐				
Positive lymph node alongside a. rectal superior	**Yes** ☐	**No** ☐				
Tumor deposits	**Yes** ☐	**No** ☐	**Number:**			
Tumor deposits diameter (mm)	**Largest:**		**Smallest:**			
Shortest tumor deposit distance to (mm)	**Positive lymph node:**		**EMVI:**	**MRF:**		
Tumor deposits visible on DWI	**Yes** ☐	**No** ☐				
**Additional comments**						
Distinguishing features, factors of insecurities and/or other noteworthy findings.						

Instructions:

1) Draw rectal cancer in all planes
2) Draw lines in the sagittal view for the high, central and low views (in relation to the tumor).
3) Mark out and number all nodal structures
4) Classify each nodal structure in the Nodal Structur Index

High

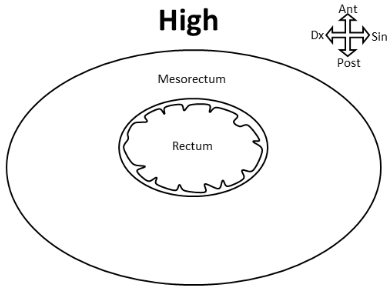

Central

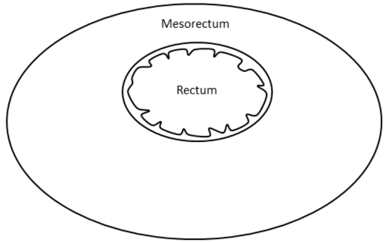

Low

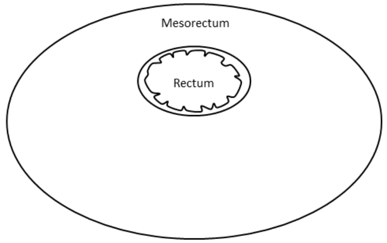

Nodal Structur Index

(LN+/LN-/TD/EMVI)

1. ______	13. ______
2. ______	14. ______
3. ______	15. ______
4. ______	16. ______
5. ______	17. ______
6. ______	18. ______
7. ______	19. ______
8. ______	20. ______
9. ______	21. ______
10. ______	22. ______
11. ______	23. ______
12. ______	24. ______

Sagittal

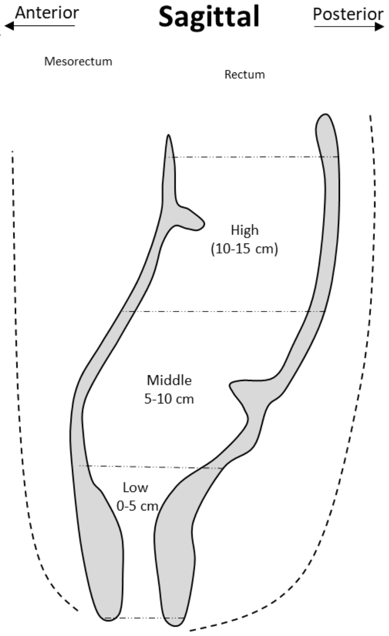

Supplement: Supplementary file 1 — ELECTRONIC SUPPLEMENTARY MATERIAL [file 330_2026_12467_MOESM1_ESM.pdf]
